# Dentists' knowledge, attitude and practice towards silver diamine fluoride therapy in Hong Kong: a mixed-method study

**DOI:** 10.3389/froh.2024.1487879

**Published:** 2024-11-19

**Authors:** Hollis Haotian Chai, Ivy Guofang Sun, Jasmine Cheuk Ying Ho, Sherry Shiqian Gao, Edward Chin Man Lo, Chun Hung Chu

**Affiliations:** ^1^ Faculty of Dentistry, The University of Hong Kong, Hong Kong, Hong Kong SAR, China; ^2^ Department of Stomatology, School of Medicine, Xiamen University, Xiamen, China

**Keywords:** silver diamine fluoride, caries, children, mixed methods research, dentists

## Abstract

This study explored the knowledge, attitude, and practice towards silver diamine fluoride (SDF) therapy among dentists in Hong Kong. The quantitative component was an online closed-ended questionnaire survey through the Society of Preventive Dentistry of Hong Kong. The qualitative component involved individual in-depth interviews with selected dentists. Descriptive analysis was performed on the quantitative data, whereas thematic analysis was performed on the qualitative data. The quantitative study invited 173 dentists, and 86 completed the questionnaire survey (86/173; 50%). All respondents were familiar with SDF and 73 (73/86; 85%) used SDF. They agreed that SDF therapy was simple (84/86; 98%), non-invasive (84/86; 98%), timesaving (82/86; 96%), effective (81/86; 94%), and painless (79/86; 92%). However, they expressed concerns about SDF's unaesthetic staining (81/86; 94%) and used it for primary teeth (77/86; 89%). The qualitative study conducted 12 individual interviews. Dentists asserted that SDF therapy is an evidence-based practice for arresting caries, including root caries. They acknowledged SDF therapy is straightforward and cost-effective. It is particularly useful for children or people with disabilities and can be used in community service. They considered the application skills, close monitoring and maintaining good oral hygiene to be vital for the success of SDF therapy. However, they noted that black staining of the carious lesions could cause patient dissatisfaction. They suggested that a guideline is warranted for SDF use. Hong Kong dentists are aware of the indications, merits and limitations of SDF therapy. They use SDF therapy for caries control, especially for children, elderly and those with disabilities, and consider it cost-effective for community dental care.

## 1 Introduction

Hong Kong is a densely populated city with a population of over 7 million people. As a Special Administrative Region of China, Hong Kong is an economically vibrant metropolis in Asia. Despite its modern infrastructure and high standard of living, dental caries is prevalent affecting individuals across all age groups. It remains an area with considerable burden to the community over decades. A prevalence of dental caries was observed in over 50% of Hong Kong preschool children aged 5 years old, with 92% of the caries remaining untreated (1). Additionally, both the adult and elderly populations experience considerable dental caries prevalence. The caries experience (in mean DMFT) of the middle-aged individuals (35–44 years old) is approximately 7, while the older adult population (65–74 years old) demonstrate a mean DMFT of 16 (2).

While traditional caries management techniques have their merits, the advent of Silver Diamine Fluoride (SDF) therapy presents a potentially more accessible, cost-effective, and minimally invasive solution (3). A bibliometric analysis revealed a significant increase in the annual production and citation counts of SDF studies in recent years (4). Notably, the highest number of SDF studies is from Hong Kong with 77 publications (4). The publications include laboratory studies, clinical trials, and reviews, which have collectively contributed to a deeper understanding of caries arrest using SDF. Furthermore, a territory-wide outreach dental service was implemented to control the prevalent dental caries in kindergarten children aged 3 to 5 years old (5). All 180,000 kindergarten children were eligible to receive SDF therapy within their respective kindergartens.

Extensive research in Hong Kong has demonstrated SDF as an effective caries management agent. There is also widespread adoption of SDF in a territory-wide community program. However, there remains a lack of qualitative data, particularly regarding dentists' perspectives on SDF. Private dental practitioners play a significant role in providing dental care, with most working independently or in small groups of general practitioners (6). The dental service system exhibits considerable flexibility. There are no government-set regulations on consultation and treatment fees, leading to noticeable variations in services and charges across different clinics (6). Examining the knowledge, attitudes, and practices of Hong Kong dentists towards SDF therapy can offer valuable insights into the potential integration of SDF into dental practices within the region. Building upon our research team's previous investigations in Japan (7) and Vietnam (8) and, this study aims to explore the knowledge, attitudes, and practices of Hong Kong dentists towards SDF therapy. Central to this study is the pivotal research question: How do Hong Kong dentists perceive SDF therapy, and what factors influence their adoption of this treatment.

## 2 Materials and methods

This mixed-method study employed the Concurrent Convergence Parallel Triangulation Design (9). It consists of a qualitative study and a quantitative study. The quantitative study was an online closed-ended questionnaire survey. Concurrent with the quantitative study, the qualitative study was conducted using individual in-depth interviews. Our research team has investigated the local dentists' perspectives towards silver diamine fluoride in Japan (7) and Vietnam (8). We adopted the methodology design to conduct a study in Hong Kong. We performed this study from August 2023 to February 2024 after ethics approval from the local Institutional Review Board (UW 23-323).

### 2.1 Quantitative study

Initially, the research team with expertise in SDF therapy and dental public health, developed an English questionnaire, which had been previously used in studies in Japan and Vietnam. The questionnaire (Table 1) consisted of four domains was adapted: (1) Dentists' knowledge; (2) Attitude; 72 (3) Practice regarding SDF therapy; and (4) Demographic information of the participating dentists. Our researchers adapted the questionnaire to fit the Hong Kong cultural context, particularly in the demographics section. Following this, two independent bi-lingual speakers proficient in English and Chinese translated the questionnaire into Chinese. To ensure semantic accuracy, another pair of independent bilingual translators back translated the Chinese version into English. After comparing the back-translated English version with the original, we made necessary revisions based on the semantic equivalence evaluation, ultimately finalizing the Chinese version of the questionnaire. The pilot study showed that 95% of dentists in Hong Kong knew about SDF. With a 5% margin of error and 90% confidence level, we needed responses from 52 dentists. Anticipating a 50% response rate, a minimum of 104 dentists had to be invited to participate in this questionnaire survey.

**Table 1:** The four domains of the questionnaire survey.

Domain 1. Dentist’s knowledge
• Have you ever heard of SDF? Yes/No
• Did you learn about SDF before you become a dentist? Yes/No
• Have you ever discussed SDF with other dentists/dental professionals? Yes/No
Domain 2. Dentist's attitude
• Which of the following would you consider as advantages of SDF?
* Please give your answer (Agree/Neutral/Disagree) to each of the following advantages*.
* *1 Simple/2 Short application time/3 Non-invasive/4 Inexpensive/5 Painless
• Which of the following would you consider as disadvantages of SDF?
* Please give your answer (Agree/Neutral/Disagree) to each of the following advantages*.
* *1 Unaesthetic/2 Unpleasant taste/3 Stains items/4 Toxicity/
* *5 Harmful—high fluoride content/6 Harmful—high silver content
• SDF can prevent dental caries. Agree/Neutral/Disagree
• SDF can arrest dental caries. Agree/Neutral/Disagree
Domain 3. Dentist's practice
• Have you ever used SDF to treat your patient?
• Are you still using SDF to treat your patient in clinic?
• How many patients did you treat by using SDF during last month? _(by number)_
• Will you use SDF to manage caries in the following situations?
* Please give your answer (always/sometimes/never) to each of the following situation*
* *1 Prevent caries in primary teeth/2 Prevent caries in permanent teeth/
* *3 Arrest primary anterior caries/4 Arrest primary posterior caries/
* *5 Arrest permanent anterior caries/6 Arrest permanent posterior caries/7 Arrest root caries
• Will you deliver SDF therapy the following populations?
* Please give your answer (always/sometimes/never) to each of the following situation*
* *1 Preschool children/2 Primary school students/3 Secondary school students/
* *4 Aged 35–64/5 Non-institutionalized older adults aged 65 or above/
* *6 Institutionalized older adults aged 65 or above/
• Will you deliver SDF therapy the following people with special needs?
* Please give your answer (always/sometimes/never) to each of the following situation*
* *1 People with mental disorders, 2 People with physical disabilities
Domain 4. Dentist's demographic information
• Main Practice (Private/Government or Institution)
○ Specialty (General/Specialist)
○ Graduate year of basic dental degree (Before 1990/1990 or later)
○ Advanced dental training or not (Yes/No)

The questionnaire was then configured into a web-based format through Qualtrics. We sent invitation to dentist-members of the Society of Preventive Dentistry of Hong Kong in October 2023 by email. The email introduced and provided aims and details of the study. A link to the questionnaire was provided to the participants and they could to respond the questionnaire in English or Chinese. Two reminders were sent to participants via WhatsApp and email. Two independent researchers compiled and compared the responses before they extracted the data into an Excel spreadsheet. After data cleaning, they conducted a descriptive analysis to evaluate the information collected.

### 2.2 Qualitative study

The in-depth interviews were conducted by a single female facilitator (HHC), who was a PhD candidate at the time of the study. She had received extensive training in qualitative research through professional courses and had a wealth of experience in conducting and publishing various qualitative studies. The participants were not personally acquainted with the facilitator prior to the interview invitation. They were provided with detailed information about the interviewer, including her credentials and the reasons for doing the research, in the introductory section of the research.

The facilitator collected dentists' perspectives on SDF therapy using a pre-established interview guide (Table 2)”. The guide encompassed four domains: (1) background information of the interviewee, and their (2) knowledge, (3) attitude, and (4) behaviors associated with practicing SDF therapy. The interviews were conducted in either Chinese or English, depending on the interviewee's preference. The facilitator took fieldnotes throughout the interviews, and audio-recorded the entire interview.

**Table 2:** Contents of the interview guide.

Key question(s)	Follow-up question(s)
Domain 1. Background information	
1.1 When did you get your basic dental training?	• How long is the basic dental training?
1.2 Which school did you study for basic dental training?	• What is your highest education level attained?
1.3 What is your current position?	• In which department do you work?
Domain 2. Knowledge	
2.1 When did you first learn about SDF?	• Why do you pay attention to SDF?
2.2 Where did you first learn about SDF?	• In what kind of curriculum did you learn about SDF? • Through what kind of media did you learn about SDF?
2.3 What SDF messages have you delivered in your professional or teaching activities?	• In what kind of curriculum have you taught SDF therapy? • What basic knowledge of SDF have you delivered? • What clinical knowledge of SDF have you delivered?
Domain 3. Attitude	
3.1 How effective do you think SDF in caries management?	• How effective do you think SDF in arresting childhood caries? • How effective do you think SDF in arresting adult caries? • How effective do you think SDF in arresting root caries? • How effective do you think SDF in caries prevention?
3.2 What are the advantages of SDF therapy?	• What are the merits of using SDF? • What are the indications of using SDF?
3.3 What are the disadvantages of SDF therapy?	• What are the limitations of using SDF? • What are the contra-indications of using SDF?
Domain 4. Practice	
4.1 How do you use SDF in clinical practice?	• With that kinds of patients would you use SDF? • How often did you apply SDF to your patients?
4.2 What were the challenges or barriers of using SDF?	• What are the clinical-related barriers of using SDF? • What are the barriers in other aspects of using SDF?

The researchers targeted dentists known for their frequent application of SDF in either clinical practice or research contexts. The selection criteria considered their clinical specialty and research focus and included professionals from both institutional and private sectors. Six dentists initially accepted the invitation to participate in in-depth interviews, forming the first cohort of interviewees. This study employed a snowball sampling strategy to recruit subsequent dentists for subsequent interviews. In each interview, the final question posed was, “Could you please recommend any dentists who might possess valuable insights regarding SDF use?” This approach facilitated the identification of additional potential participants who were knowledgeable about and experienced in SDF therapy. Throughout the study, investigators and researchers convened regular meetings to evaluate the collected data and discuss the study's progress. The sampling procedure persisted until data saturation was achieved. All invited dentists agreed to participate in the study. No repeat interviews were conducted.

Two trained researchers (HHC, JCH) transcribed the audio records verbatim and conducted a thematic data analysis. They familiarized themselves with the data by continuously reviewing interview transcripts and identifying relevant topics. They refined and sorted these topics to create a thematic framework, independently coded the transcripts, and generated a codebook for data coding through discussions and evaluations. The researchers regularly reviewed and refined codes using the codebook, revising it as needed. Finally, they summarized the data according to the constructed themes and identified connections and associations between them. The transcripts were not returned to the participants to mitigate the risk of distorting the original data. Following the separate analyses of the collected quantitative and qualitative data, the findings were compared and merged through narrative approach. During the interpretation and reporting stage, the Contiguous Approach to Integration was adopted by presenting the results in separate sections within a unified report. The fit of data integration was rigorously evaluated to check the coherence of the quantitative and qualitative findings. The methodological orientation underpinning the study appears to be a phenomenological approach and the theoretical perspective in this qualitative part is the grounded theory. The reporting of qualitative research part follows the consolidated criteria for reporting qualitative research (COREQ).

## 3 Results

### 3.1 Quantitative study

The study involved the distribution of a survey to 173 dental practitioners, with 86 completing the questionnaire, yielding a response rate of 50%. Table 3 shows 80 participants’ background information with 6 participants not disclosing their information. Forty-four respondents (44/80, 55%) were practicing in the private sector. Fifty-eight (58/80, 73%) were general practitioners. Sixty-one respondents had graduated in or after 1990 (61/80, 76%) and 63 dentists received advanced dental training (63/80, 79%). All 86 dentists were familiar with SDF, and 83 of them (83/86, 97%) had engaged in discussions regarding the topic with their colleagues.

**Table 3:** Background information of the participating dentists.

Items	Response	No. of dentists (%)
Main practice (*n* = 80)	Government/Institution	36 (45%)
	Private	44 (55%)
General dental practice (*n* = 80)	Yes	58 (73%)
	No	22 (27%)
Graduated before 1990 (*n* = 80)	Yes	19 (24%)
	No	61 (76%)
Advanced dental training (*n* = 80)	Yes	63 (79%)
	No	17 (21%)
Basic dental training in Hong Kong (*n* = 80)	Yes	47 (59%)
	No	33 (41%)
Have heard of SDF (*n* = 86)	Yes	86 (100%)
	No	0 (0%)
Have discussed SDF with other dentists (*n* = 86)	Yes	83 (97%)
	No	3 (3%)

Table 4 presents a summary of the participating dentists' attitudes on the advantages and disadvantages of SDF therapy. A high proportion of the respondents concurred that the advantages of SDF include its simplicity (84/86, 98%), time efficiency (82/86, 96%), non-invasiveness (84/86, 98%), and painlessness (79/92, 79%). The primary disadvantage identified by the dentists was the unaesthetic staining (81/86, 94%) and staining on other items (76/86, 74%). Additionally, a majority of the dentists highlighted the unpleasant taste of SDF as one of its drawbacks (64/86, 74%).

**Table 4:** Dentists’ attitudes on the advantages and disadvantages of SDF therapy (*n* = 86).

Items	Categories	No. of dentists (%)
Advantages		
Simple	Agree	84 (98%)
	Neutral	1 (1%)
	Disagree	1 (1%)
Short application time	Agree	82 (96%)
	Neutral	2 (2%)
	Disagree	2 (2%)
Non-invasive	Agree	84 (98%)
	Neutral	1 (1%)
	Disagree	1 (1%)
Inexpensive	Agree	68 (79%)
	Neutral	11 (13%)
	Disagree	7 (8%)
Painless	Agree	79 (92%)
	Neutral	4 (5%)
	Disagree	3 (3%)
Disadvantages		
Unesthetic	Agree	81 (94%)
	Neutral	4 (5%)
	Disagree	1 (1%)
Unpleasant taste	Agree	64 (74%)
	Neutral	12 (14%)
	Disagree	10 (12%)
Stains items	Agree	76 (89%)
	Neutral	8 (9%)
	Disagree	2 (2%)
Toxic	Agree	5 (6%)
	Neutral	28 (32%)
	Disagree	53 (62%)
Harmful due to high fluoride content	Agree	4 (5%)
	Neutral	13 (15%)
	Disagree	69 (80%)
Harmful due to high silver content	Agree	3 (3%)
	Neutral	22 (26%)
	Disagree	61 (71%)

Table 5 illustrates the dentists' utilization of SDF therapy in clinical care. A considerable majority of the participants (84/86, 98%) were open to employing SDF for arresting caries in primary posterior teeth, while a significant percentage (80/86, 93%) indicated a willingness to use SDF for treating root caries. Furthermore, a substantial portion of the dentists conveyed their intention to apply SDF in the treatment of caries in primary anterior teeth (75/86, 87%) and for controlling permanent posterior caries (65/86, 76%).

**Table 5:** Dentists’ practice of SDF therapy (*n* = 86).

Use of SDF therapy	Frequency of use	No. of dentists (%)
To prevent caries in primary teeth	Always	15 (17%)
	Sometimes	30 (35%)
	Never	41 (48%)
To prevent caries in permanent teeth	Always	8 (9%)
	Sometimes	33 (39%)
	Never	45 (52%)
To arrest primary anterior caries	Always	27 (31%)
	Sometimes	48 (56%)
	Never	11 (13%)
To arrest primary posterior caries	Always	38 (44%)
	Sometimes	46 (54%)
	Never	2 (2%)
To arrest permanent anterior caries	Always	9 (11%)
	Sometimes	27 (31%)
	Never	50 (58%)
To arrest permanent posterior caries	Always	17 (20%)
	Sometimes	48 (56%)
	Never	21 (24%)
To arrest root caries	Always	25 (29%)
	Sometimes	55 (64%)
	Never	6 (7%)

### 3.2 Qualitative study

**Figure 1:**
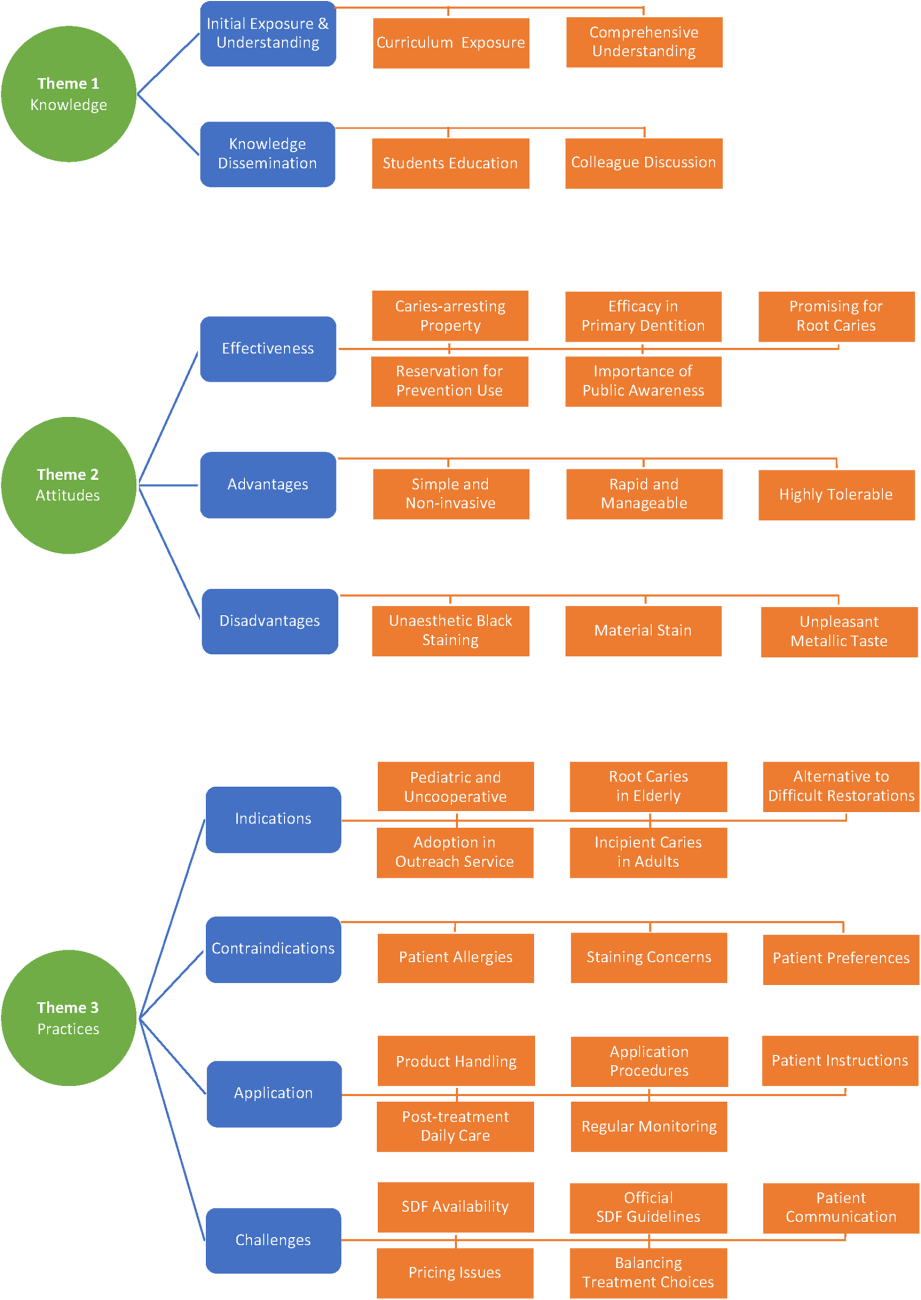
Theme tree for qualitative study (KAP towards SDF therapy among Hong Kong dentists).

The researcher facilitated 12 individual interviews and achieved data saturation. Eight of them were in-person interviews and the remaining four were telephone interviews. The researchers took field notes and audio recorded the interviews. Three themes and nine sub-themes were identified from the qualitative data. Figure 1 presents a thematic schema, consisting of three major themes and nine minor themes, derived from the qualitative study investigating the Knowledge, Attitudes, and Practices (KAP) towards SDF therapy among Hong Kong dentists.

#### 3.2.1 Theme 1- knowledges of SDF

##### 3.2.1.1 Initial exposure & understanding

The interviewed dentists demonstrated a comprehensive understanding of SDF, including its mechanism and effectiveness in arresting dental caries. Their initial exposure to SDF occurred either during their undergraduate education or advanced training, contingent upon their schooling timeline and the development of SDF in Hong Kong. The dentists identified two significant time points in the history of SDF in Hong Kong. The earliest research on SDF emerged around 2000, and more outreach programs began incorporating SDF in the 2010s.

“SDF can be utilized for arresting caries … My specialist training (2010s) involved extensive discussion on SDF application, given its growing usage amongst the dental professionals around me in kindergarten outreach programs.” —Interviewee No. 1

“The earliest research about SDF in Hong Kong, I can find this stuff from and after 2000”. —Interviewee No. 2

“I think we first learned about fluoride in year one during the PBL (Problem-Based Learning). Then, in year two, we had some lessons on caries prevention and restoration. At that time, they reintroduced us to various fluoride applications, including SDF”. —Interviewee No. 7

##### 3.2.1.2 Knowledge dissemination

Dental professionals in Hong Kong have been actively discussing the methods of SDF application and the accessibility of SDF products. Pediatric dentists possess greater experience and knowledge of SDF, frequently discussing the topic with their colleagues. Interviewed dentists involved in teaching activities have shared information about SDF with their students, based on research evidence. Presently, the curriculum at the University of Hong Kong includes the use of SDF for arresting dental caries, providing students with valuable hands-on experience in this treatment method.

“My specialist training (2010s) involved extensive discussion on SDF application, given its growing usage amongst the dental professionals around me in kindergarten outreach programs …… I think right now student also use SDF and connect to arrest carries. I will also discuss SDF with them”. —Interviewee No. 1

“The earliest research about SDF in Hong Kong, I can find this stuff from and after 2000”. —Interviewee No. 2

#### 3.2.2 Theme 2-attitudes of SDF

##### 3.2.2.1 Effectiveness

The interviewed dentists emphasize the caries-arresting property of SDF. Some of them pointed out that, in terms of arresting decay, SDF outperforms other fluoride forms due to the synergistic effect of silver and fluoride ions. For arresting caries in primary dentition, they unanimously agreed that there is sufficient evidence to support its use. As for its application in permanent dentition, particularly for root caries, they acknowledged its promising potential but emphasized the need for further evidence. Concerning the utilization of SDF as a caries prevention agent, the interviewed dentists expressed reservations due to the associated tooth staining and limited available evidence. One dentist discussed the issue at the population level, highlighting the high prevalence of caries in Hong Kong's young children and the importance of informing parents about SDF therapy as a treatment option.

“I believe we've conducted extensive research on arresting caries in primary dentition, so there's strong evidence supporting its use. As for adults, especially regarding root caries, we could use more evidence to support its use. However, the current findings are still promising” —Interviewee No. 1

“SDF can inactivate the cariogenic bacteria in the plaque, and at the same time, the fluoride can remineralize the carious lesions. So, it has two effects. Both the silver ion and the fluoride ion are actually working together to stop the decay. Because of this property, it is superior to other forms of fluoride in terms of arresting decay”. —Interviewee No. 2

“I find SDF useful for treating root caries in the elderly, as these are often hard to restore. That's why I use SDF in my practice. I'm aware SDF can prevent caries, but I seldom use it for that”. —Interviewee No. 6

“I know that in a group of very young children in Hong Kong, half of them have untreated caries. I think this information is important to tell parents that if a young child has decay, there is a choice of SDF, which is much cheaper, even though it may not look aesthetically nice”. —Interviewee No. 9

##### 3.2.2.2 Advantages

The interviewed dentists highlighted the advantages of SDF as a simple, non-invasive, and rapid treatment. They noted that it is easily tolerated by patients, particularly children, older adults, and those with special needs. The dentists also mentioned that when restorations are difficult to perform for some of the cases, SDF application remains a manageable option.

“Advantage is simple, noninvasive. We do not need any injections, and it's easily tolerated by patients”—Interviewee No. 1

“The advantages are that it's conservative, minimally invasive, and easy to use”. —Interviewee No. 4

“For the advantage, it is simple, direct. When we do the outreach, we just need to bring a tiny bottle with some applicator, then we can apply this medicine to the patients and it is very effective. It is cheap, can be readily available, particularly in the university setting”. —Interviewee No. 5

##### 3.2.2.3 Disadvantages

However, all dentists pointed out the main drawback of SDF, which is the black staining of teeth. This is especially concerning for patients with aesthetic concerns, as staining of front teeth can affect their treatment choices. Furthermore, the dentists mentioned that SDF can also stain soft tissues, such as lips and face, as well as clothing and other materials. Some dentists added that the unpleasant metallic taste of SDF may cause patients want to spit or rinse their mouths right after application, emphasizing that the applied quantity should be minimal to avoid such reactions.

“For the disadvantage. I'm quite concerned about the potential staining of the soft tissue, …… And the bad taste can be another potential disadvantage. Because when we apply the SDF, the kids may spit and rub their face together with the saliva and they will end up with some black staining over the face”. —Interviewee No. 5

“The disadvantage is the metallic taste and children dislike it”. —Interviewee No. 10

#### 3.2.3 Theme 3-practice of SDF

##### 3.2.3.1 Indications

Many dentists reported that their primary indications for utilizing SDF were primarily for pediatric patients who are too young or uncooperative, or when a primary tooth is nearing exfoliation. They also affirmed its effectiveness in managing root caries, especially during outreach services for institutionalized elderly individuals with poor oral hygiene. In adult populations, some dentists indicated that they employ SDF for incipient caries lesions to stop caries progression. Some participants also noted a higher acceptance for SDF application on posterior teeth compared to anterior teeth, emphasizing the importance of obtaining consent from the patient or caregiver before applying SDF on anterior teeth.

“I think some patients may be too young and uncooperative, or some have primary dentition with cavities. In such cases, the primary tooth might be close to the time of exfoliation”. —Interviewee No. 1

“Usually, I perform this on adults with non-cavitated caries, such as those on the enamel or early lesions. For both adults and children, I usually suggest it for caries on the molars, because it's slightly harder for them to accept application on the anterior sides”. —Interviewee No. 7

In certain cases, a dentist might choose SDF therapy for patients confronting financial constraints in treating caries lesions or for those seeking immediate caries control as a temporary solution. Furthermore, SDF therapy may be the preferred approach for medically compromised individuals who are unable to undergo conventional treatment. The participating dentists in this study highlighted the potential applicability of SDF therapy in outreach settings, particularly for patients with special needs. The interviewed dentists in Hong Kong are experienced in SDF application in their daily practice and listing the cases.

“In some cases, parents may face financial constraints in treating the lesion, and we want to buy time. In other situations, treatment may not be possible, perhaps because the child is very sick or would need to be hospitalized, making it difficult for them to come in for treatment”. —Interviewee No. 2

“SDF is highly useful for treating difficult root caries in outreach settings, especially for the elderly and special needs populations who struggle with proper oral hygiene”. —Interviewee No. 2

“For both adults and kids, if we cannot perform other treatments like fillings, then I always use SDF”. —Interviewee No. 8

##### 3.2.3.2 Contraindications

Contraindications discussed by participating dentists encompass potential allergies to SDF or concerns regarding staining and taste. Dentists in private practice highlighted the necessity of considering each patient's individual preferences and background when devising treatment plans that include SDF. Some practitioners view SDF as a first line of treatment prior to initiating restorative procedures. Meanwhile, others may continue to apply SDF during follow-up visits if the caries is arrested and cleaned well.

*“*I did apply SDF when the children were young, and as they grew up to a more understandable age, I was able to put a filling on those areas where I had applied the SDF and the lesion had been treated. So, it also made it much easier for me to implement the filling and restoration for these child patients”. —Interviewee No. 3

“I'll review a patient and determine whether to reapply the SDF or switch to another type of fluoride. Usually, I won't replace it”. —Interviewee No. 11

##### 3.2.3.3 Application

The interviewed dentists discussed SDF therapy application procedures and product handling techniques for both dentists and ancillary dental workers. They mentioned employing micro brushes for SDF application, emphasizing the importance of using minimal quantities to reduce staining and minimize the impact of the taste on the patient. Some dentists suggested that, in a clinical setting, it is essential to clean the cavity before application and control saliva. Additionally, post-application instructions were discussed, with the most frequently mentioned recommendation being to avoid eating or drinking for half an hour after treatment.

“I wouldn't say it's a challenge, but it requires careful handling because SDF is colorless. If nurses unknowingly touch it with their gloves, it may cause staining not only on the patient but also on the surrounding environment”. —Interviewee No. 8

“I find that it will be better if you clean up the cavity a bit before you apply the SDF. There's no need for drilling, but the use of a spoon excavator or air polishing would be quite a nice way to clean up the area before you apply the SDF …… Then I will give them instructions after we make the applications, he or she should not eat or drink, for the first half hour”. —Interviewee No. 12

The interviewed dentists emphasized the significance of maintaining good oral hygiene following SDF treatment. Given that caries is a multifactorial issue, they highlighted the importance of patients continuing to practice good oral hygiene and plaque control after the treatment. The dentists also noted the value of advising patients or their caregivers during the treatment process to enhance the probability of a successful outcome. Regular monitoring was deemed crucial as well. The recommended period of monitoring by the interviewed dentists is from three to six months. The combination of SDF application, professional monitoring, and daily care provided by patients or caregivers is essential for mutually beneficial results in managing caries using SDF therapy.

“I think both the application of SDF and with regular monitoring by the professionals plus the daily care of the parents or the caregiver is a very essential in a mutual benefit for the subject”. —Interviewee No. 3

“I have to review the (patient's) oral hygiene as well. If they can't clean (the teeth) well after several months, the SDF actually won't help much” —Interviewee No. 11

##### 3.2.3.4 Challenges

Many interviewed dentists cited the limited availability of SDF products in private clinics in Hong Kong as a barrier to promoting its use. Other barriers identified by the dentists include the lack of an official stance on SDF application within Hong Kong. Moreover, communication with patients or their caregivers can sometimes be challenging, as dental professionals must first manage their expectations regarding SDF therapy before application. Subsequently, effective communication is crucial to encourage patients' and caregivers' cooperation in terms of regular monitoring and maintaining good oral hygiene, which are key factors for the success of the treatment.

“I think in the clinical practice is more about if it is available in our clinic because I think not too many clinics they have the SDF”.—Interviewee No. 1

“I would say that the positives outweigh the negatives because parents are using a different standard. They focus on aesthetics, while we prioritize stopping the progression of caries”. —Interviewee No. 9

Another challenge highlighted by dentists working in the private sector pertains to the pricing of SDF treatment. In a private setting, determining the appropriate treatment to deliver involves a combination of factors. Unlike the adoption of SDF therapy in outreach programs, the choice between SDF and other restorative options in the private sector largely depends on patient age, caregiver involvement, cooperativeness, dietary control, and cost-effectiveness.

“If there's a small cavity, the choice between SDF or restoration is influenced by multiple factors. Neglecting hygiene after SDF could lead to further decay and pulpitis, causing pain and requiring more complex treatments. In private practice, the decision is influenced by a combination of factors such as the patient's age, cooperativeness, and diet”. —Interviewee No. 2

“It is hard to charge the SDF. It is hard to let the patient know the good sign of the SDF and the charge amount may not be comparable to the fillings. And for the patient's view, the fillings may be a definitive treatment”. —Interviewee No. 5

## 4 Discussion

By employing a mixed-methods approach, this research integrated quantitative survey data with qualitative interview insights to offer a comprehensive understanding of Hong Kong dentists' perspectives on SDF therapy. The value of mixed-method research stems from its capacity to merge quantitative and qualitative techniques, allowing for an in-depth exploration of complex phenomena by leveraging the strengths of both methodologies in a single study (10). By identifying the factors that influence Hong Kong dentists' adoption of SDF therapy, this study has the potential to inform dental education and public health policy, ultimately contributing to improved oral health outcomes in the region. As the global oral health community strives to address the ongoing challenge of dental caries, insights from this study can also serve as a valuable resource for other countries looking to implement SDF therapy in their dental care systems.

This study represents the first investigation into understanding the knowledge, attitudes, and practices of dentists in Hong Kong regarding SDF therapy. This research adds to a series of studies previously conducted in Japan (7) and Vietnam (8), further expanding the global understanding of SDF therapy. Hong Kong, a Special Administrative Region of China, operates under the “one country, two systems” principle. This unique system allows Hong Kong to have its independent policy on oral health care system which can be distinct from mainland China, with SDF permitted for use. The University of Hong Kong (HKU) has been deeply involved in SDF translational research for the past 20 years, making Hong Kong a significant locale for investigating SDF use in dental care. Despite the extensive research, there is a notable lack of information about the perspectives and experiences of Hong Kong dental professionals regarding SDF (11). This study endeavors to fill these gaps by implementing a mixed-method study design and methodology that was previously used in Japan and Vietnam. The strength of using a mixed-method approach lies in its ability to provide a comprehensive understanding of the subject matter. It does this by capturing both quantitative trends and qualitative insights, which together offer a more nuanced view of dentists' knowledge, attitudes, and practices regarding SDF therapy in Hong Kong.

This study presents several limitations. Firstly, the low response rate (50%) for the survey may introduce potential bias, as it could lead to findings that are only representative of dentists who have a particular interest in the subject matter and are predisposed to holding positive opinions towards SDF therapy. Despite the 50% response rate, the study's validity is maintained. The qualitative snowball sampling, while not providing a broad, generalized perspective, offers deep, nuanced insights. This strategy focuses on information-rich participants, yielding in-depth insights that counterbalance potential bias, thereby strengthening the overall conclusions of the study. Secondly, although dentists reported being fully familiar with SDF therapy, their knowledge could be further confirmed by asking questions related to SDF basics and protocols.

The knowledge of dentists in Hong Kong concerning SDF therapy reflects the development and history of SDF in the region. Limited literature on SDF in dentistry can be found before 2000. However, in 2002, we sought alternative treatments for caries management and discovered that SDF could arrest caries without the need for excavating soft, active carious tissue (12). Comparatively, the history of SDF in Hong Kong is relatively short compared to countries like Japan and Australia. SDF was developed in the 1960s by Professor Reiichi Yamaga and Professor Nishino from Osaka University, Japan (13). Dental professionals in Japan have been using SDF for over 60 years, initially for arresting Early Childhood Caries, and now it has regained popularity for addressing root caries (14). In Australia, silver fluoride (SF) has been in use since the early 1980s, following Craig et al.'s study on limiting caries progression in primary teeth (15). However, we have contributed significantly to SDF research, with 77 publications on the subject. Furthermore, SDF has been widely adopted in community service and outreach programs in Hong Kong since the 2010s (16, 17). This highlights the increasing recognition and importance of SDF as a caries management tool in various regions, including Hong Kong, despite its comparatively shorter history of use.

All participating dentists in the survey had heard of SDF, and all interviewed dentists were familiar with it. HKU, being the only dental school in the region, includes SDF in its curriculum across cariology, pediatric dentistry, and dental public health (11). Although SDF has been widely adopted in community service, hands-on experience with the treatment during basic education is reportedly lacking among the interviewed dentists. Furthermore, the interviewed dentists discussed that public education on SDF remains insufficient. As a result, many individuals may be unaware of SDF as an alternative for caries control. Addressing these gaps in practical experience and public education is essential for promoting the understanding and utilization of SDF in caries management within Hong Kong.

There is no disagreement among participating Hong Kong dentists regarding the effectiveness of SDF in arresting caries as a minimally invasive approach. Practicing dentists concur that silver and fluoride work synergistically to arrest caries (18). In Hong Kong, dentists primarily employ SDF therapy for caries control, focusing on the arrest of caries in primary teeth and root caries. A systematic review revealed an overall caries arrest rate of 81% for 38% SDF in primary teeth (19). Current evidence also supports the efficacy of a 38% SDF solution in preventing and arresting root caries (20, 21). However, some dentists noted the lack of sufficient evidence for SDF's effectiveness in preventing caries in primary teeth, a concern that aligns with the conclusions of an umbrella review (22). Additional clinical trials are warranted to ascertain SDF's role in caries prevention. This will help to further establish SDF's role in caries prevention and management within the region.

One notable issue in the practice of SDF among Hong Kong dentists is the lack of specific guidelines for indications and contraindications. Although interviewed dentists possess experience in SDF application and can identify appropriate cases in their daily practice, more detailed guidance could be beneficial. A recent discussion paper by the Asian Academy of Preventive Dentistry suggests professionally applying 38% SDF twice a year to arrest cavitated caries in increased caries risk population (23). Additionally, the “Tooth Club” website, developed by the Oral Health Education Division of Hong Kong Department of Health, suggests that SDF treatment is appropriate for children with multiple tooth decay, high risk of tooth decay, and those who cannot comply with restorative treatment (24). While SDF is widely adopted in community outreach services in Hong Kong, the situation in the private sector is more complex, as reported by the interviewed dentists. They should consider each patient's individual preferences and background when offering SDF treatment, with some choosing SDF as a first-line treatment option.

Several additional factors contribute to the successful implementation of SDF therapy mentioned by Hong Kong dentists. These factors include proper application skills and procedures, provision of post-application instructions, and effective communication with patients or caregivers. Obtaining informed consent regarding the potential for black staining is a crucial step. Moreover, dentists should consider the follow-up frequency after SDF treatment and the application frequency of SDF. While evidence suggests that biannual application of a 38% SDF solution can arrest caries by 84%, regular monitoring and follow-up application, particularly in the private sector, highly depend on patient cooperativeness (25). Finally, the availability of SDF products in the market remains a concern. Although various manufacturers offer reasonably priced options, some dentists still report a lack of availability (5). Enhancing access to SDF products would further encourage adoption and utilization in dental practices throughout Hong Kong.

## 5 Conclusion

Hong Kong dentists are aware of the merits and limitations of SDF therapy. They use SDF therapy for caries control, especially for children, elderly and those with special needs, and consider it cost-effective for community dental care. They believe that application skills and post-treatment follow-up are crucial for successfully arresting caries.

## Data Availability

The original contributions presented in the study are included in the article/Supplementary Material, further inquiries can be directed to the corresponding author.

## References

[1] Chen KJ Gao SS Duangthip D Li SKY Lo ECM Chu CH. Dental caries status and its associated factors among 5-year-old Hong Kong children: a cross-sectional study. BMC Oral Health. (2017) 17(1):121. 10.1186/s12903-017-0413-2 28859642 PMC5580282

[2] Department of Health. Oral Health Survey 2011. Hong Kong: Department of Health, The Government of the Hong Kong Special Administrative Region (2011).

[3] Wright J White A. Silver diamine fluoride: changing the caries management paradigm and potential societal impact. N C Med J. (2017) 78:394–7. 10.18043/ncm.78.6.394 29203603

[4] Jiang CM Duangthip D Chan AKY Tamrakar M Lo ECM Chu CH. Global research interest regarding silver diamine fluoride in dentistry: a bibliometric analysis. J Dent. (2021) 113:103778. 10.1016/j.jdent.2021.103778 34391874

[5] Zheng FM Yan IG Duangthip D Gao SS Lo ECM Chu CH. Silver diamine fluoride therapy for dental care. Jpn Dent Sci Rev. (2022) 58:249–57. 10.1016/j.jdsr.2022.08.001 36097560 PMC9463534

[6] Gao SS Chen KJ Duangthip D Lo ECM Chu CH. Oral health care in Hong Kong. Healthcare. (2018) 6(2):45. 10.3390/healthcare6020045 29751605 PMC6023485

[7] Chai HH Kiuchi S Osaka K Aida J Chu C-H Knowledge GS. Practices and attitudes towards silver diamine fluoride therapy among dentists in Japan: a mixed methods study. Int J Environ Res Public Health. (2022) 19(14):8705. 10.3390/ijerph19148705 35886557 PMC9319621

[8] Chai HH Dao QK Hoang TH Gao SS Lo ECM Chu CH. Knowledge, attitudes, and practices towards silver diamine fluoride among dentists in Vietnam. Dent J. (2024) 12(6):169. 10.3390/dj12060169 38920870 PMC11202812

[9] Creswell JW Clark VLP. Designing and Conducting Mixed Methods Research. Washington, DC: Sage publications (2017).

[10] Wasti SP Simkhada P van Teijlingen ER Sathian B Banerjee I. The growing importance of mixed-methods research in health. Nepal J Epidemiol. (2022) 12(1):1175–8. 10.3126/nje.v12i1.43633 35528457 PMC9057171

[11] Gao SS Amarquaye G Arrow P Bansal K Bedi R Campus G Global oral health policies and guidelines: using silver diamine fluoride for caries control. Front Oral Health. (2021) 2:685557. 10.3389/froh.2021.685557 35048029 PMC8757897

[12] Chu C Lo E Lin H. Effectiveness of silver diamine fluoride and sodium fluoride varnish in arresting dentin caries in Chinese pre-school children. J Dent Res. (2002) 81(11):767–70. 10.1177/0810767 12407092

[13] Yamaga R Nishino M Yoshida S Yokomizo I. Diammine silver fluoride and its clinical application. J Osaka Univ Dent Sch. (1972) 12:1–20. 4514730

[14] Momoi Y Hayashi M Fujitani M Fukushima M Imazato S Kubo S Clinical guidelines for treating caries in adults following a minimal intervention policy—evidence and consensus based report. J Dent. (2012) 40(2):95–105. 10.1016/j.jdent.2011.10.011 22079371

[15] Craig G Powell K Cooper M. Caries progression in primary molars: 24-month results from a minimal treatment programme. Community Dent Oral Epidemiol. (1981) 9(6):260–5. 10.1111/j.1600-0528.1981.tb00342.x 6955124

[16] Zhang W McGrath C Lo EC Li JY. Silver diamine fluoride and education to prevent and arrest root caries among community-dwelling elders. Caries Res. (2013) 47(4):284–90. 10.1159/000346620 23392087

[17] Zheng FM Lo ECM Chu CH. Outreach service using silver diamine fluoride to arrest early childhood caries. Int Dent J. (2023) 73(5):598–602. 10.1016/j.identj.2023.07.169 37612156 PMC10509413

[18] Mei ML Lo ECM Chu CH. Arresting dentine caries with silver diamine fluoride: what’s behind it? J Dent Res. (2018) 97(7):751–8. 10.1177/0022034518774783 29768975

[19] Gao SS Zhao IS Hiraishi N Duangthip D Mei ML Lo ECM Clinical trials of silver diamine fluoride in arresting caries among children: a systematic review. JDR Clin Trans Res. (2016) 1(3):201–10. 10.1177/2380084416661474 30931743

[20] Oliveira BH Cunha-Cruz J Rajendra A Niederman R. Controlling caries in exposed root surfaces with silver diamine fluoride: a systematic review with meta-analysis. J Am Dent Assoc. (2018) 149(8):671–9.e1. 10.1016/j.adaj.2018.03.028 29805039 PMC6064675

[21] Chan AKY Tamrakar M Jiang CM Tsang YC Leung KCM Chu CH. Clinical evidence for professionally applied fluoride therapy to prevent and arrest dental caries in older adults: a systematic review. J Dent. (2022) 125:104273. 10.1016/j.jdent.2022.104273 36058347

[22] Seifo N Cassie H Radford JR Innes NPT. Silver diamine fluoride for managing carious lesions: an umbrella review. BMC Oral Health. (2019) 19(1):145. 10.1186/s12903-019-0830-5 31299955 PMC6626340

[23] Zheng FM Adiatman M Chu CH Crystal YO Featherstone JD Hoang TH Recommendations on topical fluoride usage for caries management in east Asia. Int Dent J. (2024) 74:190–6. 10.1016/j.identj.2024.04.016 38871599 PMC11561476

[24] Oral Health Education Division. Tooth Club. Hong Kong: Division Department of Health The Government of the Hong Kong Special Administrative Region (2024).

[25] Yee R Holmgren C Mulder J Lama D Walker D van Palenstein Helderman W. Efficacy of silver diamine fluoride for arresting caries treatment. J Dent Res. (2009) 88(7):644–7. 10.1177/0022034509338671 19641152

